# IMPACT OF COMMUNITY TYPE ON SLEEP HEALTH DISPARITIES IN GRANDPARENTS RAISING GRANDCHILDREN

**DOI:** 10.1093/geroni/igae098.1487

**Published:** 2024-12-31

**Authors:** Melanie Stearns, Kevin McGovney, Ashley Curtis, Danielle Nadorff, Christina McCrae

**Affiliations:** University of South Florida, Tampa, Florida, United States; University of Missouri, Columbia, Missouri, United States; University of South Florida, Tampa, Florida, United States; Mississippi State University, Mississippi State, Mississippi, United States; University of South Florida, Tampa, Florida, United States

## Abstract

Grandparents raising grandchildren (GRG) report greater sleep problems than non-caregiving peers. Individuals living in low-income urban areas also report difficulty sleeping due to increased noise, light, and unsafe neighborhoods. However, no studies have examined urbanity’s impact on sleep among GRG and this study fills that gap. Grandparents living in rural (N=50, Mage=56.80), urban (N=59, Mage=53.07), and suburban areas (N=74, Mage=56.03) caring for their grandchild (>17 years old) completed the Pittsburg Sleep Quality Index (PSQI-sleep latency, sleep duration, sleep efficiency, sleep medication, sleep disturbances, and global sleep scales) and indicated their community type (rural, urban, suburban). Two-way ANOVAs evaluated interactions (sleep x community) and main effects. Pairwise comparisons clarified significant differences. Interactions were not significant. There was a significant main effect (p=.047) of community on sleep medication use, sleep disturbances (p=.009), and global sleep problems (p=.012). Urban grandparents reported more sleep medication (M=1.58+\-1.20) than rural (M=1.06+\-1.27; p=.046) or suburban grandparents (M=1.07+\-1.26; p=.026). Urban grandparents reported more sleep disturbance (M=1.63+\-.64) than rural (M=1.25+\-.55; p<.001) or suburban grandparents (M=1.44+\-.53; p=.080). Urban grandparents reported more global sleep problems (M=10.61+\-3.17) than rural (M=8.28+\-3.43; p=.010) or suburban grandparents (M=8.61+\-2.87; p=.010). Results suggest that level of urbanity impacts sleep health disparities among GRG and urban GRG may be at greater risk for sleep problems. More research (e.g., longitudinal) should examine what contributes to increased sleep problems among urban GRG, but these results suggest that behavioral sleep treatment programs should potentially target environmental and socioeconomic factors unique to urban GRG (e.g., increased noise, decreased safety and security, etc.).

